# Enriching the lives of children with acquired brain injury and their caregivers: experiences from peer mentorship sports camps

**DOI:** 10.3389/fresc.2024.1285742

**Published:** 2024-05-31

**Authors:** Pia Wedege, Silje Mæland, Anestis Divanoglou, Frank Eirik Abrahamsen

**Affiliations:** ^1^ Department of Sport and Social Sciences, Norwegian School of Sport Sciences, Oslo, Norway; ^2^ Department of Follow-up Services After Spinal Cord Injury, Sunnaas Rehabilitation Hospital, Bjørnemyr, Norway; ^3^ Department of Global Public Health and Primary Care, University of Bergen, Bergen, Norway; ^4^ Department of Rehabilitation Medicine and Department of Medical and Health Sciences, Linköping University, Linköping, Sweden

**Keywords:** peer mentorship, peer support, acquired brain injury, sports camp, Active Rehabilitation, qualitative study, children, caregivers

## Abstract

Peer-based community interventions have shown promise in improving health management and fostering coping skills and psychosocial functioning among individuals with a disability. Active Rehabilitation camps are examples of peer-based community interventions that provide structured, time-limited peer mentorship in conjunction with sports and leisure activities. These camps hold potential benefits for individuals with acquired neurological injury. However, the specific impact of Active Rehabilitation camps on children or individuals with acquired brain injury remains unexplored. In this longitudinal, qualitative study, we explored children with an acquired brain injury and their caregivers' experiences with an Active Rehabilitation camp in Norway through observations and interviews with nine children and ten caregivers. Using an abductive thematic analysis, we identified an overarching theme: Active Rehabilitation peer mentorship camps enrich the lives of children with acquired brain injury and their caregivers. The theme contains three sub-themes: (1) Interacting with peers made me wiser, (2) Nudging from peer mentors made me feel better, and (3) A sense of companionship through meeting peers. Peer mentorship, sports and leisure activities, and the safe camp atmosphere benefitted children with acquired brain injury and their caregivers. The children gained knowledge, motivation, and self-worth, and their caregivers had greater impetus to prioritize their children's independence. Meeting peers and peer mentors led to friendships and sustained social connections. The Self-Determination Theory was of assistance in explaining the informants' experiences. Active Rehabilitation camps provide children with acquired brain injury and their caregivers with an opportunity to develop better coping skills, improve psychological functioning, and build more robust social networks.

## 1 Introduction

The United Nations Convention on the Rights of Persons with Disabilities states that peer support should be provided to help individuals with disability achieve full inclusion and participation in all aspects of life (1). Additionally, the World Health Organization (WHO) recommends integrating peer support into time-limited self-management courses to enhance the health management skills of people with disability (2). Time-limited peer-based community interventions for individuals with acquired brain injury (ABI) or spinal cord injury can improve coping skills and psychosocial functioning (3), which aligns with the WHO's goal of enhancing community-based rehabilitation for people with disability (4).

ABI is an umbrella term for various brain injuries, whether traumatic or non-traumatic (5–8). Related impairments may be severe, persistent, and sometimes life-long and may affect neurocognitive, psychological, and physical functioning (5, 9–13). Age at injury plays an essential role in how the individual is affected. When damage occurs in childhood and adolescence, the brain development process may be disrupted (14), and the child's ability to learn new skills may be impacted, leading to difficulties functioning at home, at school, and in their local community (15). Hence, an ABI affects the child and their family (5).

Active Rehabilitation (AR) is a global community, peer-based rehabilitation model developed in Sweden in the late 1970s (16). Training camps are the most common activity among AR organizations (16). These camps are structured and time-limited, leveraging sports and leisure activities to enable individuals to realize their full potential in skill development and participation by enhancing independence in their daily lives and boosting self-esteem (17). Peer mentorship, defined as intentional, purposeful, and unidirectional peer support provided by designated peer mentors (18, 19), is an essential element of AR camps (16). Further, a peer mentor “possesses experiential knowledge of a specific behavior or stressor and similar characteristics as the target population” (20).

Implementing theoretical frameworks in research on peer support interventions may help explain and synthesize results across studies (18, 21). Self-Determination Theory [SDT; (22)] is among several motivational theories suggested as suitable for research on peer support interventions (3, 23–25). SDT is an organismic approach to understanding how biological, social, and cultural conditions promote or hinder inherent capacities for psychological growth, commitment, and wellness (22). According to SDT, humans are viewed as active and growth-oriented, seeking the necessary nutriments to integrate themselves into their surrounding social structures and experience a fuller, more enduring, and profound sense of well-being (26).

As a meta-theory encompassing several mini-theories (22, 27), three specific mini-theories are particularly relevant to the current study. These include the Basic Psychological Needs Theory (26), Organismic Integration Theory (28), and the Relationships Motivation Theory (29). Basic Psychological Needs Theory identifies three basic psychological needs: autonomy, competence, and relatedness, which are the core of SDT (22, 26). The satisfaction of these needs is crucial for individuals to achieve eudemonic well-being (22). SDT also discusses a continuum of motivation and regulatory styles, where Organismic Integration Theory outlines different types of external motivation, ranging in various levels of autonomy. In summary, the higher the autonomy, the more likely the individual is to experience well-functioning (22, 28). In Relationships Motivation Theory, SDT further discusses essential elements of high-quality relationships, such as mutual involvement and being oneself (29). SDT's philosophy aligns well with the aims of AR camps and can, therefore, assist in explaining the findings of research on these camps.

While there is emerging evidence of AR camps' effects on adults with spinal cord injury (30–33), there is no evidence of AR camps' impact on children or individuals with ABIs (3). Furthermore, the caregiver's perspective concerning participation in AR camps has never been explored. Hence, we aimed to explore the experiences of children and youths and their caregivers participating in an AR camp for individuals with ABI. More specifically, the current study aimed to explore the following questions:

1. What are children's and youths' experiences with AR camps?
2. How do caregivers perceive their children's experiences with AR camps?
3. What are the caregivers' experiences with AR camps?

## 2 Materials and methods

### 2.1 Study design

This qualitative, longitudinal study was approved by the Norwegian School of Sport Sciences' Ethical Committee (Ref. no 229–160622) and the Norwegian Centre for Shared Services in Education and Research (Ref. no 521550). The study's conduction and reporting were informed by guidelines from Tracy (34) and the consolidated criteria for reporting qualitative research (COREQ) (35).

### 2.2 Methodological orientation

This study takes a critical realist approach that integrates a realist ontology (the belief in a real world existing independently of our thoughts and perceptions) and a constructivist epistemology (the idea that our understanding of the world is constructed from the researchers' and the study participants' perspectives and standpoints) (36, 37).

### 2.3 Setting

The Sunnaas Foundation is a Norwegian non-profit organization that runs AR camps in Norway (38). The Sunnaas Foundation's AR camp for children with ABI is called Brain Camp Yng [BCY; (39)]. Initiated in 2019 and run annually (38), the camp is free of charge and hosts self-referred mentees (39). The staff comprises peer mentors and non-disabled assistants, the latter being primarily healthcare professionals. BCY aims for children to challenge themselves in sports and leisure activities with peers and peer mentors, learn from each other, and build a network of peers (39). A description of BCY following the Template for Intervention Description and Replication [TIDieR; (40)] can be found in the Supplementary Material S1.

BCY 2022 took place in August, and 13 children and youths (mentees) with ABI attended. Usually, family members do not attend AR camps, but BCY allows each mentee to bring a caregiver. Hence, 13 caregivers (parents and grandparents) participated in BCY 2022. The mentees were divided into a children's group (6–12 years) and a youth group (13–16 years). Each was matched with a peer mentor based on their goals for camp (e.g., practice using the affected hand), shared experiences related to their impairment(s) (e.g., sensitivity to noise and light, fatigue), or difficulties encountered in their local community (e.g., school settings, friendships; personal communication with Brain Camp Project Coordinator, June 2023). BCY 2022 adhered to the ten key elements of AR camps [Table 1; (16)].

**Table 1:** Description of the 10 key elements of Active Rehabilitation camps in relation to Brain Camp Yng 2022.

10 key elements of Active Rehabilitation camps	Description of Brain Camp Yng 2022 (BCY 2022) in relation to the 10 key elements of Active Rehabilitation camps
(1) Peer mentors	At BCY 2022, nine persons with ABI (17–33 years) were enrolled as mentors. Except for one mentor, all had participated as mentors in one or more previous camps. One mentor was part of BCY's organizing committee and oversaw the mentors. Each mentor (together with a non-disabled assistant) was assigned to one or two mentees.
(2) Non-disabled assistants	In total, 15 non-disabled assistants with healthcare backgrounds (six physical therapists, one occupational therapist, four sports therapists, two nurses, one medical doctor, and one psychologist) were present throughout the camp, many of whom had experience working with individuals with ABI. The non-disabled assistants did not receive specific training before attending the AR camps. Two had not previously participated in AR camps. Most non-disabled assistants (and the mentors) were assigned to one or two mentees throughout the camp, forming groups of three or four, including a mentor, a non-disabled assistant, and one or two mentees. Furthermore, the non-disabled assistants were assigned to the children or youth group, and one was part of the BCY's organizing committee.
(3) Activities of daily living and skills training	Training in activities of daily living (ADL) was not part of the formal schedule but was adapted to the needs of each mentee. In these sessions, the mentees received help and guidance in ADL from mentors and non-disabled assistants. Caregivers were encouraged to limit their assistance in ADL during the camp. ADL training may include getting dressed, showering, eating, planning the equipment needed for the activities, transfers, and walking.
(4) Sports and leisure activities	In BCY 2022, the following activities were included: water acquaintance/swimming, kayak/canoe, stand-up paddling, archery, boxing/self-defense, cycling, orienteering, yoga, air pistol shooting, fishing, virtual reality (VR) games/art, overnight camping (youth group only), art sessions (children group only), and meeting alpacas (children group only). Each session lasted about 1.5–3 h, with two to five sessions offered per day (Supplementary Material, S2). External professional instructors led most activity sessions in close cooperation with the mentors. The daily program started at 8 am with a camp dance and finished around 9:30 pm. The caregivers had a different schedule and occasionally participated in activities with their children.
(5) Education	In BCY 2022, the children and youth groups engaged in sessions with the psychologist and mentors to discuss coping strategies. Caregivers participated in three sessions: two with the psychologist and medical doctor and one with the mentors. The topics of the sessions with the psychologist and medical doctor included “getting to know each other” by sharing their children's history and “coping strategies.” In the mentor session, the mentors told their stories and the caregivers could ask questions.
(6) Training environment	BCY 2022 was based at Vestre Kjærnes Gård (kjaernes.no), a conference center on a farm southeast of Norway. It is a rural location with a lake nearby, facilitating water sports activities. All camp attendees were accommodated at the farm: children shared a room with their caregivers, youths shared a room with another mentee, and the youths’ caregivers had private rooms. All meals were served in the barn, where three tables were set up: one for the children group, one for the youth group, and one for the caregiver group, encouraging discussions and interactions. Mentors and non-disabled assistants joined the groups to which they were assigned. In one corner of the barn was a small area for relaxation, comprising two sofas and a table with drawing and art material and board games. Most sports and leisure activities were held in the outdoor area surrounding the farm. The yoga, VR, and education sessions were held in a meeting room and the farm's living room.
(7) Admission criteria	The admission criteria for BCY 2022 were as follows: children and youths between 6 and 16 years old, diagnosed with ABI, and able to walk or use a manual wheelchair (sunnaasstiftelsen.no). Eligible first-time mentees were prioritized. Three mentees at BCY 2022 had attended previous camps.
(8) Setting goals, initial and final assessment	The organizing committee contacted mentees before the camp to learn about their needs and camp goals and provide camp information. The information received from the mentees and their caregivers was used to customize the camp experience. During camp, staff meetings for the mentors and non-disabled assistants were held each evening. Mentees’ progress, daily routines, and logistics were discussed in these meetings to prevent mistakes and adjust the support provided to mentees. At BCY 2022, there was no initial or final assessment.
(9) Training of peer mentors	The eight mentors in BCY 2022 applied to the Sunnaas Foundation to participate, seven of whom had attended the Sunnaas Foundation's Peer Mentor Training Program (Sunnaasstiftelsen, n.d.). This program consists of four courses per year, each lasting three to four days, covering topics such as defining the mentor role, setting goals, motivation, and communication.
(10) Duration of AR camps	BCY 2022 lasted six days, from July 31st to August 5th. Mentors and non-disabled staff arrived one day before the mentees to become acquainted and finalize the camp program's organization.

ABI, acquired brain injury; AR, Active Rehabilitation; ADL, activities of daily living.

### 2.4 Study participant selection

We invited all mentees and their caregivers to participate in this study if BCY 2022 was their first AR camp experience. Before BCY 2022, camp organizers contacted mentees and caregivers and informed them of the study on behalf of the research team. Upon arriving at camp, the mentees and caregivers interested in the study could discuss it further with the first author (PW) and provide informed consent. Caregivers consented for themselves and their children. The project information offered to eligible study participants was age-adapted.

### 2.5 Data generation

#### 2.5.1 Observations

SM and PW participated in the camp as non-disabled assistants, which enabled them to perform field observations and discuss these observations daily. SM followed the children's group all week, while PW moved between groups and participated in part of the caregivers' schedule (see Supplementary Material S2, for BCY's schedule). Digital recordings were made recurrently using a cell phone throughout the day and transcribed each night. The template used for the field observations was based on Lareau's (41) guidelines. Field notes were not analyzed separately, but PW used this information when interviewing mentees and caregivers after camp. Attending the camp as staff also enabled PW to build rapport with the study participants before the interviews, as recommended by Eder and Fingerson (42) and Boylan et al. (43). Furthermore, by attending the camp, SM and PW could observe how mentees were affected by their ABI.

#### 2.5.2 Interviews

We used semi-structured interview guides (Supplementary Material S3) pilot tested on children within the age range and mentees and caregivers from previous camps. This approach allowed us to investigate central themes of BCY's focus areas, such as self-esteem, activity, participation, and relationships, and delve deeper into the study participants' psychological needs fulfillment as suggested by SDT. Incorporating the principles of SDT into our interview guides not only enriched our study with a theoretical framework but also allowed us to capture the unique experiences of our study participants. PW conducted all interviews, either in-person or digitally, according to guidelines for conducting interviews with children and adolescents (42, 43). Mentees and caregivers were interviewed twice, first immediately after the camp and again after approximately six months. Mentees and caregivers decided the time and place of the interviews (see Table 2 for information about the interviews). In the interviews, PW used pictures from the camp as prompts and picture cards in case mentees struggled to describe situations or feelings. Six mentees chose to have their caregiver(s) present, which was optional, as were any breaks needed during the interview. Hence, some caregivers attended four interviews: two with their child and two alone. The caregivers provided all injury-related information in the first interview, and a log was recorded after each interview. The interviews were recorded digitally and transcribed verbatim by PW. All mentees and caregivers were offered to review their transcribed interviews, with only one caregiver choosing to do so.

**Table 2:** Information about study participants and interviews.

	Mentees (*n* = 9)	Caregivers (*n* = 10)
Personal characteristics		
Gender	2 girls/7 boys	5 females/5 males
Age (years)		
Range	7–16	31–70
Mean (SD)	12 (3)	47 (10)
Median (Q1–Q3)	11 (10–13)	48 (41–50)
ABI characteristics		
Etiology	3 traumatic, 6 non-traumatic (incl. encephalitis, stroke, cancer)	
Age at injury (years)		
Range	0–15	
Mean (SD)	7 (5)	
Median (Q1–Q3)	6 (3–10)	
Time since injury (years)		
Range	1.5–11.1	
Mean (SD)	5.2 (3.1)	
Median (Q1–Q3)	4.0 (3.5–6.5)	
Interviews (first/second)	*n* = 9/8	*n* = 10/9
Place of interviews (1^st^/2^nd^)		
Interviewees’ home	9/6	8/6
Official meeting room	0/1	0/1
Digital	0/1	2/3
Time of interview after camp (1^st^/2^nd^)	1–13 days/5.5–7 months	1–13 days/5.5–7 months
Length of interview (1^st^/2^nd^) (minutes)		
Range	17–49/6–17	23–50/12–32
Mean (SD)	28 (10)/10 (4)	35 (9)/19 (5)
Median (Q1–Q3)	24 (20–34)/9 (7–11)	31 (28–44)/19 (18–20)

ABI, acquired brain injury; SD, standard deviation; Q, quartile.

### 2.6 Analysis

We analyzed the data using an abductive thematic analysis (44, 45). Our understanding of abduction is that prevailing theories (i.e., SDT) partly influenced our foci in the semi-structured interview guides and, more so, our interpretation of the results. In the analysis, we followed the eight steps outlined by Thompson (44): (1) PW transcribed all interviews verbatim, and FEA and PW familiarized themselves with the data through multiple transcript readings. (2) Using semantic codes, PW performed the initial inductive coding in MAXQDA 2022 (46). The coding was done inductively, trying to bracket our knowledge of SDT. Still, this knowledge may have influenced the coding process. (3) PW and FEA then discussed the different codes' meanings and situations for use. (4) PW and FEA used a code matrix to gather codes into categories and possible themes. The entire research team further reviewed the codes, categories, and themes and examined the relationships among these to ensure essential data were captured. The transcripts, initial codes, categories, and themes were discussed among the research team several times to ensure rigor. (5) We drew on our knowledge from similar studies and theoretical motivational frameworks, such as SDT, when discussing the results. (6) The research team explored similarities and differences within and between the mentee and caregiver groups. (7) The research team attempted to display visually how the themes were derived from the initial codes and categories (Table 3). (8) The write-up included a description of the method, study participants, setting, and results with illustrative quotes and a discussion. Quoted mentees and caregivers were identified by “Child_no”, “Youth_no”, or “Caregiver_no” for identity protection. Because many of the younger mentees struggled to express themselves and give thick descriptions, we deemed it necessary to paraphrase and summarize many of their quotes to describe their experiences better. Hence, most quotes are from the caregivers.

Tracy (34) advocates honesty and transparency concerning researchers' biases to improve the quality of qualitative research. As such, this study's research team consisted of three physiotherapists (two female and one male) and one sports psychologist (male), three of whom had experience with AR camps within or outside Norway. After BCY 2022, SM became head of research at the Sunnaas Foundation. PW and SM were among the staff at BCY 2022, and PW had attended two BCY camps before 2022 and several other AR camps in Norway. Spending time with all mentees and caregivers during BCY 2022 allowed her to build rapport with study participants. Hence, when the interviews started, she was already familiar with them, potentially rendering mentees' and caregivers' camp experiences more accessible. PW's and SM's camp experiences may have affected their data interpretation. However, the research team was mindful of this possibility and thoroughly discussed codes, themes, and analyses to ensure rigor and credibility.

## 3 Results

This section will share information about the study participants and their camp reflections. An overarching theme evolved through iterative analyses of the mentees' and caregivers' reflections and discussions among the research team: Active Rehabilitation peer mentorship camps enrich the lives of children with ABI and their caregivers. The overarching team was created from three sub-themes: (1) Interacting with peers made me wiser, (2) Nudging from peer mentors made me feel better, and (3) A sense of companionship through meeting peers. Each sub-theme consisted of two or more categories (Table 3).

**Table 3:** Overview of themes, categories, and codes.

Overarching theme	Sub-themes	Categories	Codes
Active Rehabilitation peer mentorship camps enrich the lives of children with ABI and their caregivers.	Interacting with peers made me wiser	Managing fatigue	Fatigue
			Pain
			Epilepsy
		Gaining valuable insight	Sports technique
			Assistive devices
			Challenges of ABI in adolescence
			Adapted sports
			Variations of ABI
	Nudging from peer mentors made me feel better	Motivation	Physical activity
			Becoming a peer mentor
			Camp participation
			Local support systems
			Independence in ADL
		Mastery and confidence	Physical activity
			Self-esteem
			Self-confidence
			Social confidence
	A sense of companionship through meeting peers	Interacting with peers	Friendship
			Network of peers
		Interacting with peers and peer mentors	Relatedness
			Feelings of trust, honesty, comradery, understanding, support, acceptance
			Nuanced perspectives/normalization of the situation
		Interacting with peer mentors	Promote openness about ABI
			Hope

ABI, acquired brain injury; ADL, activities of daily living.

Color codes: Yellow = mentees, Blue = caregivers, Green = mentees and caregivers.

Throughout our analysis, we highlighted similarities and differences within and between the mentee and caregiver groups and noted changes between the first and second interviews. A peer is often defined as someone of equal standing to others, whether in, i.e., age or experience. Therefore, within the context of BCY, the mentees' peers were both fellow mentees and peer mentors. The caregivers' peers were the other caregivers.

### 3.1 Description of study participants

Nine of ten eligible mentees and all ten eligible caregivers accepted the invitation to participate in the study (see Table 2 for study participants' characteristics). During the second interview, one mentee and one caregiver from the same family could not participate due to health issues. While most mentees exhibited minimal visible physical impairments, they faced challenges with fatigue, memory, concentration, epilepsy, and sensitivity toward noise and light.

### 3.2 Interacting with peers made me wiser

This sub-theme describes how interactions with peers and peer mentors assisted mentees and caregivers in developing better insight and establishing management strategies for fatigue and other ABI consequences.

#### 3.2.1 Managing fatigue

Many mentees struggled with fatigue due to their ABI, and both mentees and caregivers were given information and gained a better understanding of fatigue at camp. Mentees discussed fatigue during a session with the psychologist and peer mentors and had the opportunity to observe the peer mentors and fellow mentees managing their fatigue during camp. Many learned about different ways and the importance of rest, as reported by one mentee: “I learned sometimes to take breaks, even when I want to continue to do something” (Child_9, second interview). Gaining more information about fatigue and participating in the intensive camp schedule led one mentee to challenge their physical limits and taught them that physical exhaustion is not a matter of worry. Having discussed fatigue with the peer mentors, one mentee expressed the intent to adjust rest habits upon returning home, acknowledged the significance of informing their local community about their need for rest, and felt empowered to start a dialog with their school about how to accommodate their need:

Hmm, it is very important to inform those closest to me, like at school, to inform the teachers that I sometimes need breaks. “Sometimes you guys need to help me decide when I should rest because sometimes, I struggle to figure it out myself.” Uh, I had a meeting with the school yesterday, and we decided that initially, it's thirty minutes on and thirty minutes off, and then we can gradually increase or decrease it. […] And I have never really considered informing others about the issues I’m dealing with, but when they [peer mentors] mentioned it, I realized how important it is for them to know why I might need to take a break during class. And I told my teacher and asked him to inform all the teachers and all the students about it. (Youth_3, first interview)

In the follow-up interview, some mentees said they managed their fatigue better and incorporated better rest routines at home. One youth described using physical activity as a respite from the headaches suffered as a consequence of ABI. Caregivers confirmed that their children had developed a more profound comprehension of fatigue by observing fellow camp attendees:

He may have learned to take a short break beforehand […], and it's vital that he sees that others also need a break. I think it's very important for him to see that it's not just him who needs breaks. (Caregiver_4, first interview)

The caregivers did not have a dedicated session to discuss fatigue, but many reported gaining knowledge of the importance of rest and how to help their children rest. Some found it beneficial to observe how other caregivers carefully structured their days to manage their children's fatigue, having received little assistance from their local community in dealing with this issue. Others reflected on difficulties differentiating their children's fatigue and lack of motivation, well described by one caregiver: “What is fatigue and what is just being a normal child who doesn’t want to go on a hiking trip?” (Caregiver_3, first interview).

Many caregivers were initially concerned about the busy camp schedule, as they were explained the importance of rest upon their children's initial injury. However, as the camp progressed, they noticed their children coping well and realized they could push them further:

Hmm, we have definitely learned and seen how important rest is and how it's something everyone needs. […] Although we've been told it's a common need, and we see it in [child's name], it's kind of reassuring to know that there's some truth to it for the broader group of brain-injured individuals. Um, so that's one thing, and on the other hand, it's also good to push a little too, uh-huh. […] We have noticed at the camp that pushing a little can be helpful too. (Caregiver_1, first interview)

During the initial interview, some caregivers intended to prioritize a balance between activity and rest for their children. During the follow-up interview, many reported successfully maintaining this focus. At camp, some caregivers learned that high physical activity levels could positively impact secondary impairments such as pain and epilepsy. Despite encountering challenges in achieving the right balance between rest and activity and differentiating fatigue from physical exhaustion and lack of motivation, they felt less anxious about their children's activity level after the camp.

#### 3.2.2 Gaining valuable insights

In addition to knowledge about secondary impairments, some mentees claimed to have learned about the variety of ABI, how to perform sports activities, and how sports can be individually adapted to their needs.

According to the caregivers, their own and their children's understanding and awareness of ABI improved after meeting peers and peer mentors. Many emphasized the significance of the peer mentors sharing their experiences regarding potential challenges during adolescence, and some gained advice from other caregivers about handling challenging situations, useful devices (e.g., noise-canceling systems), and appropriate physical activities for their children. Further, some caregivers elaborated on how they acquired a better understanding of variances that exist across the nation in local support systems. After learning more about ABI, some caregivers recognized that their responses and feelings toward the injury were reasonable, given the circumstances. Others posited that this newfound knowledge of ABI inspired them to exercise more patience with their children. During the subsequent interview, some caregivers verified that they had indeed become more composed and understanding with their children.

Um, it has probably influenced me a little. Specifically, in those situations where things are moving slowly or when [child's name] forgets things, it's like, I don't get frustrated with the situation; it's okay, that's just how it is. Yeah, I think it is a part of it [ABI], and I've become a bit calmer about, you know, things will be okay eventually, yeah. (Caregiver_1, second interview)

### 3.3 Nudging from peer mentors made me feel better

This sub-theme describes how nudging from peer mentors and engaging in sports and leisure activities increased mentees' and caregivers' motivation and mentees' confidence and sense of mastery.

#### 3.3.1 Motivation

Just over half of the mentees were physically inactive before attending the camp, and many expressed interest in attempting new sports and leisure activities. Mentees mentioned their fun trying various activities when asked what they remembered best from the camp. They expressed a desire to try more activities in the future, with some sharing specific activities they wanted to pursue. The caregivers confirmed this desire to continue certain sports and leisure activities, and a few made detailed plans. Others said their children were already engaged in physical activities and thus refrained from making further plans. In the follow-up interview, some mentees previously uninvolved in sports or regular physical activity stated they had either begun participating in planned sports or increased their physical activity.

Some mentees expressed being motivated by their encounters with peer mentors, with whom they could relate and compare themselves. One youth expressed that it was comforting and motivating to know that one peer mentor also had a paralytic arm and added that having someone who could relate to their condition was helpful and inspiring. After attending the camp and bonding with the peer mentors, some youths endeavored to become peer mentors themselves, and one even disclosed having enrolled in a peer mentor training program. The motivation was to support and encourage other children and youth who had experienced ABI. Almost all mentees were inspired to participate in more camps, and all stated they would recommend BCY to other children with ABI.

A few caregivers observed that the children displayed increased motivation to improve their independence in ADL after interacting with peers and peer mentors:

He learned something from them [peer mentors]. He understood that you can be really ill, but through training and dedication, you can manage to live an independent life. That's what he sees, and that's his goal. And in a way, it gives him a little push forward. (Caregiver_2, first interview)

Some caregivers admitted being overly protective of their children, hoping to prevent further hardships or challenges. They realized that although their intentions were good, this approach could harm their children's growth and development and hamper mastering activities. The caregivers explained that at camp, they discovered their children's potential to become more independent in ADL, with the camp's focus and peer mentors gently pushing mentees being essential to this increased awareness. Several caregivers expressed a desire to expand their children's independence and set higher expectations upon returning home:

Ever since we returned from camp, I have, in a way, let go a little. I can't be such a helicopter mom. I have to, and of course, [child's name] enjoys it when Mom serves and helps and follows him around the room and all those things, but maybe I've become a bit more like, “No, [child’s name], you have to do it yourself” [laughter]. Of course, if he’s tired, I still do those things, but maybe I've picked up some tools from the peer mentors and staff about the importance of training independence. And you don't achieve that without doing things yourself. So yes, from personal care to moving around the house, we encourage [child's name] to master it himself, uh-huh. (Caregiver_2, first interview)

While not universally reported in follow-up interviews, some caregivers noted that their children had become more independent.

Many caregivers reported difficulties collaborating with their children's schools and local support teams for the necessary help and adaptations. They explained that interactions with the other caregivers and peer mentors at camp motivated them to persist in ensuring optimal outcomes for their children.

#### 3.3.2 Mastery and confidence

Many mentees expressed a sense of mastery when discussing various activities. This experience brought them joy and a valued feeling of accomplishment:

And knowing that even though you have a brain injury, it doesn't mean that you are incapable of doing most things. You can do almost anything anyone else can do. This is a valuable lesson to keep in mind. (Youth_3, second interview)

In the follow-up interview, one youth reported feeling empowered to try new activities at home after mastering new skills at camp. The peer mentors' encouragement to try the various camp activities was well-received by mentees, who appreciated the peer mentors' ability to understand their limitations and when to push them. Some mentees also noted a shift in their self-esteem, self-confidence, or social confidence following camp, with one explaining that it was easier to talk to people and that they had become more outgoing after camp.

Many caregivers hoped their children would gain mastery during camp and reported success. They credited the mentees’ mastery to the peer mentors' gentle encouragement and support, instilling belief in the mentees' abilities:

And that's the take-home message: how skilled the peer mentors are in getting the mentees not just to believe that they can do things on their own but also proving it to them. I think that's super important, and I would have liked to have more examples. But it’s like, “Oh, am I going kayaking?” “Oh no, can I row without that floater on the kayak?” “Yes, you can!” And they [peer mentors] take you, they give you that little push, which is so valuable, and you bring that confidence with you. In that sense, their role is absolutely priceless, right? Because they [peer mentors] know, they know that they [mentees] can do it because they have been in the same position. They [peer mentors] know that the kids have much more capacity than they believe. (Caregiver_2, first interview)

The caregivers appreciated the so-called “you can do it” attitude at camp and believed the camp had positively influenced their children's self-esteem and confidence. They attributed the changes to interacting with and feeling acceptance from peers. They explained these positive changes could be observed in the mentees’ attitudes, moods, and postures:

I can tell that he is, how should I put it, much happier now. Um, yes, towards the end of the camp, I noticed that he was walking with an upright posture, whereas he usually walks like this [demonstrates a stooped posture], slightly leaning forward, looking down, and hesitant to make eye contact with people. It seems like he's now talking to people and laughing. (Caregiver_4, first interview)

### 3.4 A sense of companionship through meeting peers

This sub-theme describes how mentees, caregivers, and peer mentors connected at camp, how these meetings with peers and peer mentors were perceived, and what thoughts and feelings were facilitated by these meetings. Before attending camp, few mentees and caregivers had individuals with ABI in their network. Some caregivers felt isolated in their communities and feared their children struggled with loneliness.

#### 3.4.1 Interacting with peers

The primary camp goal for many mentees and caregivers was for mentees to connect with peers and form friendships. Virtually all mentees expressed gratitude for meeting new people at camp and making friends. With these interactions, they felt enjoyment, alleviation of their loneliness, that they were not alone in their struggles, and that having an ABI was not something abnormal. The fellow mentees were perceived as being understanding. Moreover, the mentees appreciated not having to explain the injury and its consequences repeatedly and enjoyed meeting someone in whom they could see themselves:

They [peers] understand a bit better and things like that, I think, uh-huh […] Yes, uh-huh. I don't have to explain about fatigue and things like that. […] It made a difference to be at a camp with people who have brain injury compared to a camp where nobody has brain injury. There's quite a big difference […] because they are, like, similar in a way, there are people who resemble me, who also have injuries. (Youth_1, second interview)

Connecting with other mentees was challenging for some due to age differences, but most expected to stay in touch after the camp. The youth group established a social media group, and follow-up interviews revealed that the group persisted, though involvement varied. Some of the children communicated with one another, either via phone or online gaming.

Most caregivers perceived their children had a positive experience meeting peers at the camp. They believed their children had felt accepted and included, made new friends, and received encouragement from the group. For some, this contrasted with their home situation, where the children struggled to maintain friends post-injury. The caregivers related this to the children's behavioral change after the ABI, which friends found difficult to handle. Some caregivers said that their children constantly compared themselves with their schoolmates at home, diminishing their self-esteem. Hence, introducing the children to others with ABIs was beneficial for their comparative behaviors. According to the caregivers, the children gained insight into how others handled similar situations, became more comfortable discussing their condition and difficulties, and developed a more nuanced perspective of a typical child's daily life. Some caregivers observed that their children encountered difficulties engaging with other mentees due to their varying levels of physical impairments.

Several caregivers expressed an interest in attending the camp to establish connections with other caregivers. Following the camp, many maintained these relationships. Some caregivers noted that while they did not frequently utilize this network, they viewed it as an easily accessible resource when needed. Upon meeting fellow caregivers at camp, they quickly noted a strong sense of trust and honesty within the group. The caregivers also expressed feelings of support, camaraderie, and a sense of belonging to a community. They described the camp as a safe place to be vulnerable, where it was effortless to share experiences of having a child with an ABI. Further, they valued encountering others who could empathize with their situation rather than offer unsolicited advice.

It has been a week of real highs and lows; there has been joy and tears and the opportunity to talk with others who understand what you're talking about instead of sitting there with your friends and trying to wrap your thoughts in a certain way. The opportunity to talk with other parents and just open that lid and know that they truly understand the emotions you've been dealing with or the thoughts you have, right? To gain an understanding that, as my child puts it: “you are not alone”. It's a different kind of understanding, you know. (Caregiver_8, first interview)

#### 3.4.2 Interacting with peer mentors

The children perceived the peer mentors as friendly and supportive but were unsure of their specific roles. The children's caregivers confirmed this lack of awareness but also claimed that their children had positive experiences with and quickly formed bonds with the peer mentors. Some caregivers perceived that when meeting peer mentors who could relate, their children were encouraged to be open about their injuries, participate more, and take on challenges during camp activities.

The youths perceived the peer mentors as individuals who motivated them to participate in various activities, educated them on how to manage living with an ABI, and assured them through their expertise and experience:

You don't feel different because there are people who either struggle with the exact same thing or face similar challenges. And it provides a sense of security, knowing they have experiences and knowledge that can make you wiser. (Youth_3, second interview)

In addition, a few mentees noted that the peer mentors were present not out of obligation but because they genuinely wanted to assist.

The youths' caregivers perceived that their children admired the peer mentors. In addition, the caregivers believed the peer mentors encouraged the youths' independence, had faith in their abilities, comprehended their daily challenges, and assisted them in recognizing that their experiences and reactions were typical for their circumstances.

Caregivers commended the peer mentors for their credibility, uniqueness, and importance in enhancing the camp experience. They described the peer mentors' encounters as inspiring, impressive, and motivating and reported the belief that peer mentors genuinely cared about their children. The caregivers listened attentively to the peer mentors' narratives and valued their candidness and transparency regarding their harsh experiences with ABI. When asked about their interaction with the peer mentors, one caregiver expressed their appreciation:

I thought it was really nice. They [peer mentors] truly gave of themselves despite their challenges and tough lives. It was truly, perhaps, the most wonderful experience […] because they could articulate their struggles and show their emotions. But still, they persevere and share so much. Um, I saw that there were a lot of tears and painful emotions but also many beautiful things. So, I think it must have been quite exhausting and tough for them as well. (Caregiver_1, first interview)

When asked if camp participation affected the caregivers' outlook for their children's future, some identified no change, either due to already existing high expectations or to challenges related to their children's diagnoses. Others said that when their child suffered the ABI, they felt all plans and visions for their child's future crumble. Thus, interacting with the peer mentors and observing how they managed life with an ABI instilled hope for and a more optimistic perspective of their children's future: “When I see the peer mentors, it feels good, it is perhaps easier to see a future for my child” (Caregiver_4, second interview). By interacting with the peer mentors, the caregivers also accepted that having an ABI did not mean their children's lives would be less, as expressed by one caregiver when asked what they had learned: “Well, it was never to give up or underestimate yourself. Life isn’t over even though you struggle with fatigue or have a disability. Yes, I experienced the joy of life being with them [the peer mentors]” (Caregiver_2, second interview).

## 4 Discussion

This longitudinal, qualitative study explored the experiences of children with ABI and their caregivers who participated in a structured, time-limited peer mentorship sports camp. To our knowledge, this is the first qualitative, longitudinal study exploring these individuals' experiences with an AR camp. From the analyses, we constructed three sub-themes: (1) Interacting with peers made me wiser, (2) Nudging from peer mentors made me feel better, and (3) A sense of companionship through meeting peers, from which an overarching theme was constructed: Active Rehabilitation peer mentorship camps enrich the lives of children with ABI and their caregivers.

In this overarching theme, we emphasize the peer mentors' essential role in supporting mentees and caregivers by listening, encouraging, prompting, and empathizing. Moreover, we found that by interacting with peers and peer mentors, mentees and caregivers gained more insight and knowledge of ABI and its consequences, changing their behavior and everyday lives to improve coping. Mentees gained mastery through activities and developed motivation for physical activity and participation, and caregivers also noted a positive shift in mentees’ self-esteem and self-confidence after the camp. Caregivers increased their desire to focus on improving their children's independence in ADL, with some managing to maintain this focus and improve their children's independence after camp.

Mentees and caregivers built relationships with peers during camp, which continued after the camp. They expressed gratitude for meeting people who shared their experiences and truly understood their situations, as these connections enabled camaraderie and belonging among mentees and caregivers and helped ease feelings of isolation. Due to these interactions, caregivers also reported greater optimism about their children's futures. Further, the camp's sports and leisure activities, the safe social atmosphere, and the “you can do it” attitude adopted at BCY facilitated and enforced mentees' and caregivers' experiences.

### 4.1 Increased insight and overprotective caregivers

The mentees and their caregivers gained valuable insights about ABIs and related impairments at camp, which corresponds with other studies on structured, time-limited peer mentorship activity camps for individuals with acquired neurological injuries (47–50). In BCY, caregivers were encouraged to allow their children to manage ADL (such as meals) independently, and the children often participated in camp activities without their caregivers present. According to Grolnick and Apostoleris (51), when parents experience uncertain environments (e.g., their children having an ABI), they tend to become more controlling. Moreover, caregivers may be overprotective of and indulge their children due to the fear and relief associated with the child surviving a life-threatening injury or accident (8). Similarly, the caregivers in our study explained that their inclination toward overprotection stemmed from a desire to prevent undue hardship for their children. Several caregivers commented that increased knowledge of ABIs changed their behavior toward their children. They became more patient and understanding, provided less assistance in ADL at home, and paid greater attention to structuring daily schedules to accommodate their children's fatigue.

According to Organismic Integration Theory, the continuum of extrinsic motivation ranges from external regulation, through introjected and identified, to integrated regulation (28). Within the introjected regulation type, behavior is not entirely externally motivated but regulated by internal pressure, such as feelings of contingent self-worth (22). Ryan and Deci (52) discuss how parents might undermine autonomous motivation, increasing the introjection regulation of anticipated behaviors (i.e., through guilt and shame). The data in the current study do not indicate parental pressure; instead, they suggest a tendency toward minimal exposure to choices and challenges by being overprotective. Significantly, during camp, caregivers acquired a deeper understanding of ABIs, developed a heightened awareness of their children's capabilities, limitations, and requirements, and became more adept at discerning appropriate moments for encouragement and boundary establishment (possibly reflecting caregivers' need for competence satisfaction). By enhancing caregivers' understanding and knowledge of ABIs through the camp, basic psychological needs were met, leaving caregivers more inclined to give children greater freedom and allow them to explore and develop autonomy.

### 4.2 Sports and leisure activities as a facilitator

A lack of role models, assistive equipment, and facilities hinders physical activity among children with disability (53). Hence, attempting sports and leisure activities at camp with peers and peer mentors, combined with the opportunity to use suitable adaptive equipment and techniques, offered new experiences and enhanced the likelihood of mentees engaging in sports and leisure activities upon returning home.

Children with chronic illness sometimes cannot enroll in summer camps due to their impairments or because they need close medical attention (54). In BCY, healthcare professionals are among the camp staff, enabling children needing close medical attention (e.g., due to epilepsy) to participate in activities without a caregiver. Many camps for children with disability aim to allow mentees to play and be “normal” children (55). Our finding corroborates this, as mentees shared that they were given respite from managing the injury at camp.

The fun activities were central to both the children's and youths' positive camp experiences, a finding supported by other studies exploring diagnosis-specific camp experiences (47, 56–58). This perception of fun may have supported the mentees' tolerance for the intense camp schedule. SDT describes engaging in activities out of pure joy as an intrinsic motivation, which depends on experiences of autonomy, competence, and relatedness (22, 59). Ryan and Deci (22) state that the experience of competence satisfaction depends on positive feedback and optimal challenges, which is “the match of persons’ abilities with task demands” (22). Hence, although identifying ideal camp activities and adapting these to a broad range of ages and impairments can be challenging (47, 56), it is vital for competence satisfaction and to ensure a reasonable challenge for everyone. Feedback focusing on competence is more likely to support intrinsic motivation, while feedback leading the person to feel critiqued or controlled can reduce intrinsic motivation (22). Moreover, verbal persuasion from significant others and vicarious experiences are sources of self-efficacy (60, 61), acted upon by the peer mentors at BCY by gently nudging and persuading the mentees to try activities, praising them for their efforts, and showing joy when the mentees partook in activities. Furthermore, the peer mentors demonstrated how they, with their impairments, completed tasks related to ADL and sports throughout camp. Mentees' experiences of mastery in the sports and leisure activities at camp may have motivated them to continue physical activity engagement after camp.

Children and youths with ABIs may experience low self-esteem (8, 62), and sports activities and peer interaction at camp seemed to boost their self-worth. A sports setting, such as the sports and leisure activities at BCY, may be considered a “natural context” and hence facilitate conversations between peer mentors and mentees (42) about complex topics, such as their injuries, impairments, and social lives. Thus, sports and leisure activities may facilitate personal growth.

### 4.3 Networks and the need for relatedness

Meeting peers, whether other children, youths, caregivers, or peer mentors, was an essential part of our study participants' camp experience. As confirmed in our study, difficulties in establishing and sustaining friendships and loneliness can occur after an ABI (8). We believe the goal of making friends at camp and achieving this reflects the need for and satisfaction of relatedness, as described in SDT (22). In SDT, relatedness concerns feeling socially connected and is experienced when one feels part of a group, cared for, and important to others (22, 29). Mentees in the present study described that at camp, they felt they belonged to a group that understood their struggles, made friends, and were seen, looked after, and cheered on by peer mentors, experiences supported by Analytis et al. (47).

In the present study, contact within these networks of peers was somewhat limited after camp. However, some caregivers explained that even though they did not use the network much after camp, they still felt part of a group that cheered them on and argued this network would be easy to reach out to. This perception of available support may lead to a more positive assessment of stressful events and better coping skills (63).

According to Relationships Motivation Theory, high-quality relationships are facilitated by autonomous motivation, i.e., the willingness to participate in the relationship (29). Similarly, mentees and caregivers perceived that the peer mentors participated in the camp because they wanted to help. Moreover, they were genuinely happy for mentees when they mastered activities or social settings during camp. Furthermore, experiencing oneself contributing to others is essential to satisfying the need for relatedness (22). Hence, the autonomous motivation in these relationships between mentees and peer mentors may satisfy the need for relatedness and enhance the well-being of both parties.

Differences in age and injury impairment and trajectories were perceived to hamper bonding within the camp's mentee and caregiver groups and with the peer mentors. Matching mentees with mentors based on personal and injury characteristics were some of the matching criteria used in BCY 2022 and is common in peer mentorship interventions (64–66). However, Standal (67) argues that pure mirroring of injuries or demographics is insufficient to facilitate peer mentorship, as the empathy required of a peer mentor is more related to the ability to place themselves in the mentee's position. Furthermore, a supportive environment is, among others, characterized by “effectance supporting” (22). Hence, if mentees feel that peers or peer mentors are too well-functioning compared to themselves, they will not experience a sense of mastery, and competence will be thwarted. Our findings argue for condition-specific camps with a broad range of impairments and ages among mentees and peer mentors. Moreover, it seems essential that peer mentors manage their injuries well enough to motivate caregivers and mentees to continue facing their challenges and, simultaneously, are not perceived as super-humans.

### 4.4 Study strengths and limitations

Rather than relying solely on adult perspectives to understand the lives of children and youths, it is recommended to incorporate their voices in research concerning ABI to provide a platform for their thoughts and interpretations (42, 43). While following guidelines for interviewing children with ABI (43), we encountered challenges related to both ABI-related impairments and general interactions with children.

To establish rapport, study participants were free to select the interview locations (68), and children and caregivers chose interview times, sites, and attendees. Most opted for home interviews, enabling a familiar environment, but this choice brought distractions from siblings, family members, and pets. Caregivers were present in some children's interviews, aiding responses with prompts, but some children deferred to caregivers, leading to lessened engagement. Further, in three caregiver interviews, the children were present, potentially inhibiting open sharing.

Camp attendance by PW and SM helped build rapport and an understanding of how the children's impairments might implicate the interviews. However, building rapport with children takes time (68), and although PW comprehended the mentees' impairments, an even more profound level of familiarity would have been helpful.

To address fatigue, we kept sessions concise and provided breaks, including activities like games and sports, to maintain rapport. In alignment with guidelines (43), PW allowed the children control of the recording equipment to foster trust and used visual aids, including pictures, to address verbal comprehension and memory issues. Yet, although such prompts are recommended to enhance comprehension (43), we occasionally found that the children failed to concentrate on the questions and became more interested in looking at the people in the pictures. Although open-ended questions are advised when exploring peoples’ experiences and perceptions (69), children might prefer closed-ended questions due to verbal limitations (68). Our comfort-led conversations yielded brief answers, subsequently relying on caregivers' insights.

Incorporating children, youths, and caregivers enabled a diverse experience exploration, and analyzing within and across groups strengthened the study's results. Study participant recruitment was exhaustive, and data saturation was not addressed due to study constraints.

Methodological orientations and the context bound our findings. Qualitative research offers context-rich knowledge that is transferable via analytical generalization (70, 71), and our study may benefit similar peer mentorship camps for various conditions. In addition, quantitative approaches to complex interventions, such as peer mentorship camps, are challenging due to ethical and logistical reasons and to ensure measuring the right outcomes (54, 72). Instead, we employed a qualitative design supported by a longitudinal format. It is possible that an extended follow-up period and inclusion of study participants who attended more than one camp would have revealed additional behavioral changes or offered insights into cumulative effects. Finally, exploring peer mentors' perspectives and age-based mentee differences could further enrich our understanding of AR peer mentorship camps.

### 4.5 Conclusion

Despite the short duration, participating in BCY seemed to contribute to valuable knowledge and experiences gained by children and youths with ABI and their caregivers. The overarching theme, “Active Rehabilitation peer mentorship camps enrich the lives of children with ABI and their caregivers”, reflects peer mentors' essential role at BCY in facilitating knowledge gain, personal growth, and network building. Furthermore, this theme incorporates the sports and leisure activities offered at the camp and the safe and encouraging atmosphere, which appear vital to the mentees' and caregivers' experiences. The mentees expressed that they encountered a supportive environment that fostered enjoyment, mastery, motivation, and self-worth, all central facets of SDT (22). By connecting with peers and peer mentors, mentees learned to manage their ABI while forging meaningful friendships.

In addition, caregivers found great value in the support network formed among themselves, offering them a deeper understanding of ABIs and valuable perspectives on the experiences of those affected. Their newfound insight spurred positive changes in their parenting approaches, including increased patience, reduced assistance with ADL, and a more structured daily routine to accommodate their children's fatigue. Furthermore, meeting peer mentors gave them a more positive outlook on their children's future. The enhanced confidence of caregivers in their children could potentially foster a more autonomy-supportive parenting approach. Over time, this could nurture the children's need for autonomy and, perhaps indirectly, their need for competence as the increased trust in their abilities is demonstrated.

According to Ryan and Deci (26), individuals who prioritize meaningful relationships, personal growth, and community contribution—all linked to basic psychological needs—tend to experience greater eudemonic well-being. Therefore, by providing a safe and supportive camp atmosphere that fosters friendships, an increased understanding of ABIs, and self-worth, mentees and caregivers may be inspired to make positive changes in their daily lives and engage more fully with their local community in pursuit of eudemonic well-being.

## Data Availability

The datasets presented in this article are not readily available due to data protection regulations in Norway. Our research data falls under a category that requires strict confidentiality and cannot be openly shared. However, we have taken measures to fully anonymize the interview transcripts to ensure the privacy and anonymity of the participants. These anonymized transcripts can be made available upon request to the corresponding author.
