# Supplementary material for: Enriching the lives of children with acquired brain injury and their caregivers: experiences from peer mentorship sports camps
Source: Front Rehabil Sci. 2024 May 31;5:1285742. doi: 10.3389/fresc.2024.1285742 (PMC11176490; doi:10.3389/fresc.2024.1285742)
Supplement: Supplementary file 1 [file Datasheet1.docx]

Supplementary Material

**Enriching the Lives of Children with Acquired Brain Injury and their Caregivers: Experiences from Peer Mentorship Sports Camps**

Pia Wedege, Silje Mæland, Anestis Divanoglou, Frank Eirik Abrahamsen^*^

*** Correspondence:** Frank Eirik Abrahamsen: frankea@nih.no

# Supplementary Data

**(S1) Template for Intervention Description and Replication (TIDieR) for Brain Camp Yng – Active Rehabilitation camps for children and youths with acquired brain injury and their caregivers**

1. **Brief name**

Brain Camp Yng (BCY). An Active Rehabilitation (AR) camp for children and youths with acquired brain injury (ABI) and their caregivers.

1. **Why**

Research indicates that community peer-based interventions benefit individuals with acquired injuries and provide an opportunity to interact with others with similar disabilities. AR camps are structured, time-limited activity camps providing face-to-face peer mentorship. BCY targets children and youth (6-16 years) with ABI and their caregivers. The camps allow for formal and informal interactions with peers and peer mentors and provide opportunities for physical activity, acquiring new knowledge, and creating a network of peers. BCY is led and delivered by peer mentors and healthcare professionals, who, as a team, are called leaders. The peer mentors are credible resources and living examples of what mentees can achieve and can convey to mentees examples of how to handle their condition.

1. **What material & 4. What procedures**

AR camps offer sports and leisure activities to improve function and independence and introduce regular practice. Activities depend on locally available facilities, including swimming, kayak/canoe, stand-up paddling, archery, boxing/self-defense, cycling, orienteering, yoga, air pistol shooting, fishing, overnight camping, and art sessions. The activity sessions typically last 1.5-3 hours each, with two to five daily sessions. Peer mentors lead some activity sessions, but external professional instructors teach most.

In AR camps, training in activities of daily living (ADL) is incorporated into the daily schedule as needed, with the help and guidance of peer mentors and healthcare professionals. The caregivers are encouraged to limit their assistance during the camp. ADL training may include getting dressed, showering, planning the equipment needed for the activities, transfer training, and walking.

Psychoeducation sessions aim to help mentees and caregivers gain knowledge, share information, share their challenges, receive support, and learn how to manage ABI effectively. Mentees attend one or two sessions led by a psychologist and peer mentors, while caregivers attend approximately three sessions led by a psychologist and medical doctor or peer mentors. The session topics vary based on the attendees' needs and may cover fatigue, coping strategies, and managing adolescents with ABI.

At a behavioral level, BCY aims to improve motivation to be physically active, boost self-esteem and self-efficacy, and cultivate a positive attitude toward one's ability to participate in society and maintain a social life.

1. **Who provided**

The Sunnaas Foundation, a non-profit organization, runs AR camps in Norway, including BCY.

*Peer mentors* have a lived experience of ABI and are included in the camp as organizers, trainers, educators, and role models. Those with an ABI who want to become peer mentors at BCY apply to The Sunnaas Foundation. Some peer mentors have participated in earlier camps. The peer mentors are paid, and the peer mentor-mentee ratio is approximately 1:2. One peer mentor is part of the BCY organizing committee. The Sunnaas Foundation runs a peer mentor training program ([Sunnaasstiftelsen - Vil du bli mentor?](https://www.sunnaasstiftelsen.no/vil-du-bli-mentor)), but it is not a requisite to have participated in this program to become a peer mentor at BCY. The peer mentor training program consists of four courses within a year, each lasting three to four days. The program includes topics such as defining the roles of peer mentors, setting goals, motivation, and communication.

*Non-disabled assistants* support the peer mentors and mentees during the AR camps. The non-disabled assistants are healthcare professionals experienced in working with people with disabilities (e.g., physiotherapists, occupational therapists, sports therapists, psychologists, nurses, and medical doctors). The non-disabled assistants do not receive training before attending the camps. Most non-disabled assistants are assigned to one or two specific mentees throughout the camps. One non-disabled assistant is part of the BCY’s organizing committee.

The peer mentors and non-disabled assistants assist the sports instructors in adapting the activities to each mentee.

1. **How**

BCY is a face-to-face residential camp. Structured and informal sessions are primarily group-based and allow for some flexibility for peer mentors to individualize training/activities and individual contact. The mentees are divided into two groups: one for mentees aged 6-12 (Children group) and one for mentees aged 13-16 (Youth group). Peer mentors and non-disabled assistants are assigned to the Children or the Youth group. The caregivers are in a separate group.

*Sports and leisure activity* sessions are time-limited and allow mentees to practice individually or in groups, depending on the activity. Mentees are encouraged to try all activities and challenge themselves. The sessions include demonstrations, mirroring instructions, strategy provision, and progressive challenges tailored to each mentee.

*Psychoeducation* sessions involve formal group sessions.

Both sports and leisure activity and psychoeducation sessions involve positive reinforcements and encouragement, camaraderie, and persuasion (e.g., “If I can do it, you can do it”). The sessions aim to reinforce motivation, mastery, and self-efficacy. The last camp dinner is organized as a formal dinner/party. During this dinner, mentees, peer mentors, caregivers, and non-disabled assistants are encouraged to entertain. In addition, the organizing committee provides each mentee with a speech and a card highlighting the positive qualities observed by peer mentors and non-disabled assistants throughout the week. The purpose of this gesture is to enhance the mentees' self-confidence and encourage them to persist in pursuing their goals.

1. **Where**

BCY 2022 is located at Vestre Kjærnes Gård [(kjaernes.no)](https://www.kjaernes.no/). This is a conference center located on a farm southeast of Norway. It is a rural location with a lake nearby, facilitating water sports activities. All camp attendees are accommodated at the farm. The children share a room with their caregivers; the youths share a room with another mentee; the youths’ caregivers have private rooms. All meals are served in the barn, where three tables are set up: one for the Children group, one for the Youth group, and one for the Caregiver group. This setup facilitates interactions within the separate groups and promotes independence during meals. The peer mentors and the non-disabled assistants join their assigned groups. In one corner of the barn is a small area for relaxation, containing two sofas and a table with drawing and art material and board games. Most sports and leisure activities are outside in the surrounding area. The psychoeducation sessions take place in the farm’s living room. Outside the barn is a small area furnished with sofas and chairs, where camp attendees gather in between activities and in the evenings.

1. **When and how much**

BCY camp lasts six days. Peer mentors and staff arrive one day early to finalize the program and get to know each other. At 07:45 every morning, the program commences with a camp dance. The program concludes at 21:00 for mentees and caregivers. For peer mentors and non-disabled assistants, the program ends at 22:00 after assessing the day and devising a plan for the following day.

1. **Tailoring**

The mentees self-refer to The Sunnaas Foundation. Their application provides information about injury etiology, functional mobility, and reasons for participation in the camp. After being accepted for participation, the organizing committee has a videoconference with the mentee and caregiver, where the organizing committee receives more information about the mentee’s impairments and healthcare needs, and the mentee’s specific camp goals are discussed. These goals are further discussed with the mentee and caregiver when they arrive at the camp. During daily camp staff meetings, healthcare needs, goals, and necessary accommodations are discussed and modified as needed.

1. **Modifications**

Changes in the planned schedule may change due to weather conditions.

1. **How well – Planned**

The planning of BCY adheres to the 10 key elements of AR camps (Divanoglou et al., 2017), with some modifications due to the ABI diagnosis compared to the spinal cord injury diagnosis and due to the age range at BCY. 1) A peer mentor-mentee ratio of at least 1:5. 2) Trained non-disabled assistants. 3) ADL sessions. BCY does not incorporate specific wheelchair skills training. 4) Sports and leisure activities incorporated in the schedule on a daily average of at least two hours. 5) Education sessions. 6) Training environment in the community. 7) Specific admission criteria (i.e., diagnosed with an ABI, no severe infections, being able to walk with or without assistance, or using a manual wheelchair). 8) Goal setting is part of the camp. 9) Peer mentors have received formal training in AR. 10) The duration of the camps is between five to ten days.

1. **How well – Actual**

Mentees and caregivers are encouraged to participate in all physical activity and education sessions. However, some mentees may only attend part of the sessions to accommodate ABI impairments or other requirements.

# Supplementary Tables

(S2) Table 1: Mentees’ schedule BCY 2022

| **Time** | **Sunday 31^st^ of July** | **Monday 1^st^ of August** | **Tuesday 2^nd^ of August** | **Wednesday 3^rd^ of August** | **Thursday 4^th^ of August** | **Friday 5^th^ of August** |
| --- | --- | --- | --- | --- | --- | --- |
| 07:45 |  | Morning dance | Morning dance | Morning dance | Morning dance | Morning dance |
| 08:00 |  | Breakfast | Breakfast | Breakfast | Breakfast | Breakfast |
| 09:00-10:30 |  | Youths: Canoe/kayak  Children: Air pistol shooting | Youths: Boxing  Children: Yoga | Youths: SUP  Children: Fishing, swimming, beach activities | Youths: Packing up the camping site and rest  Children: Cycling | Outdoor activities, archery |
| 10:45-12:15 |  | Youths: Air pistol shooting  Children: Canoe/kayak | Youths: Yoga  Children: Boxing |  |  | Meeting with evaluation and camp movie |
| 12:30-14:00 |  | Lunch and rest | Lunch and rest | Lunch by the beach and rest | Lunch and rest | Lunch, check-out, and departure |
| 14:00-15:15 | Arrival, check-in, meet with MD and nurse, peer mentors, and staff | Youths: Orienteering  Children: Art/VR | Youths: Archery  Children: Meet alpacas | Youths: Swimming, beach activities, starting to set up the camping site  Children: SUP | Youths: Cycling  Children: Art project outside |  |
| 15:30-16:45 |  | Youths: Art/VR  Children: Orienteering | Youths: Workshop with psychologist and peer mentors  Children: Archery |  |  |  |
| 17:00-18:30 | Dinner and rest | Dinner with information meeting and rest | Dinner with information meeting and rest | Dinner with information meeting and rest | Rest and preparation for Farewell dinner and party |  |
| 18:30-20:30 | Welcome to BCY, ‘Get-to-know-each other’ activities |  | Youths: Fishing  Children: Workshop with psychologist and peer mentors | Youths: Workshop with peer mentors around the campfire  Children: Movie, board games | Farewell dinner with entertainment by staff, mentees, and carers |  |
| 20:30-21:00 | Light snack | Light snack | Light snack | Light snack |  |  |
| 21:00 | Good night/Meeting for camp leaders | Good night/Meeting for camp leaders | Good night/Meeting for camp leaders | Good night/Meeting for camp leaders | Good night/Meeting for camp leaders |  |
| Abbreviations: MD= medical doctor, VR=virtual reality, SUP=stand up paddle | | | | | | |

# Supplementary Data

# (S3) Interview guide - children/youths

## Background

- Can you please tell me a little about yourself and your family?
  - School
  - Hobbies
  - Siblings/parents
  - Injury and help in ADL

## Camp

- Please tell me why you wanted to participate in the camp.
  - Camp expectations?
  - Camp goals?
- Please tell me your most memorable camp experience(s).
  - Negative experiences?
  - What did you like the most?
- Have you experienced anything like the camp before?

## Self-image

- How would you describe yourself?
  - Would you have described yourself differently before the injury?
  - Has this changed after the camp?

## Relationships and peer support

- Did you know anyone with an ABI before attending the camp?
  - Is being in touch with peers with ABI in your daily life essential?
- How was it to meet the other mentees at camp?
  - Do you expect to keep in touch with the other mentees from the camp?
- How was it to meet the peer mentors?
  - What do you think the peer mentors did at camp?
- What did you learn from the other mentees or peer mentors?

## After camp

- How was it to return home after the camp?
- Please tell me something you learned at camp that will be useful for your daily life.
- Is there anything you would like to do/start with after camp?

# Interview guide - caregivers

## Background

- Please tell me about yourself
  - Family
  - Work
  - Education
- How does your child’s ABI impact your daily life?

## Camp

- Please tell me why you and your child wanted to participate in BCY
  - Camp goals
  - Camp expectations
- Please tell me about your camp experiences
  - Your most memorable camp moment
- How do you think your child was affected by camp participation?
- Please tell me what you learned at camp
  - What do you think your child learned?

## Relationships and peer support

- Please tell me about your social network in your daily life
  - Is anyone in a similar situation?
- Please tell me about your experiences of meeting the other caregivers at camp
  - Do you expect to stay in touch after camp?
- Please tell me about your experiences of meeting the peer mentors at camp
- How do you think it affected your child to meet the other mentees/peer mentors?

## After camp

- Please tell me how it was to return home after camp
- How do you expect camp participation to affect your daily life?
- Do you expect to behave differently toward your child after camp?
- In what way has camp participation affected your outlook on the future?

**
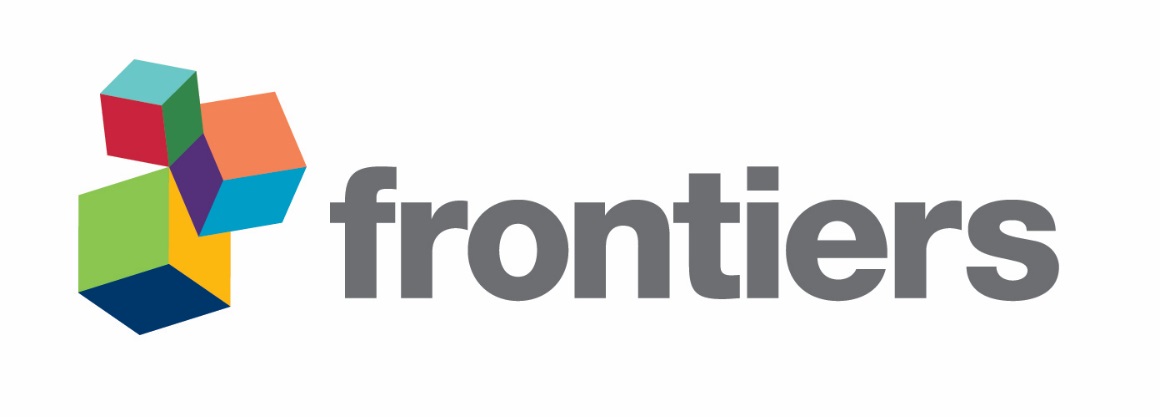
**
